# Differential Diagnosis Value of Shear-Wave Elastography for Superficial Enlarged Lymph Nodes

**DOI:** 10.3389/fonc.2022.908085

**Published:** 2022-06-30

**Authors:** Yanjuan Sun, Wen Wang, Chengrong Mi, Qian Zhang, Kun Zhang

**Affiliations:** ^1^ Department of Ultrasound, General Hospital of Ningxia Medical University, No. 804 South Shengli Street, Yinchuan, China; ^2^ Department of Ultrasound, Cardiovascular and Cerebrovascular Disease Hospital, General Hospital of Ningxia Medical University, Yinchuan, China; ^3^ Central Laboratory and Department of Medical Ultrasound, Shanghai Tenth People’s Hospital, Tongji University School of Medicine, Shanghai, China

**Keywords:** ultrasound, shear-wave elastography, superficial enlarged lymph nodes, differential diagnosis, conventional ultrasonography

## Abstract

**Objectives:**

To evaluate the diagnostic efficiency and diagnostic threshold of conventional US and shear-wave elastography (SWE) in superficial enlarged lymph nodes (LNs).

**Methods:**

A total of 204 patients with superficial enlarged LNs were enrolled in this retrospective study aged 46.0 ± 15.2 years from March 2020 to March 2021. LNs with a long axis larger than 0.7 cm were considered as superficial enlarged. Before the histological biopsy, LNs that were considered suspicious according to both conventional US and SWE were included, while LNs with no or unclear pathological results, or with no satisfactory SWE images, were excluded. The conventional and 2-D SWE examinations were performed with Aplio i800 and Acuson sequoia equipped with i18LX5 linear-array transducer (5-18 MHz) and 10L4 linear-array transducer (4-10 MHz), respectively. Both E Median and Vs Median parameters were investigated by two senior ultrasound physicians. The pathological results were performed as the gold standard.

**Results:**

Variables including transverse axis size, lymphatic hilum, L/T ratio, echogenicity, and color Doppler pattern were considered significant. The mean E Median value in benign, metastatic LNs, and lymphoma were 28.26 ± 8.87 kPa, 77.46 ± 22.85 kPa, and 50.37 ± 5.41 kPa (*p <*0.001), while Vs Median values were 3.02 ± 0.50 m/s, 4.87 ± 0.90 m/s, and 4.09 ± 0.22 m/s, respectively (*p* < 0.001). The diagnostic performance indicated the high sensitivity, specificity, PPV, NPV, and overall accuracy of conventional US combined with SWE. The optimal cutoff values of E Median and Vs Median for predicting malignant LNs were 42.90 kPa and 3.73 m/s, respectively. As AUC value, sensitivity, specificity, accuracy, PPV, and NPV revealed, the indexes of E Median were 0.976, 0.927, 0.975, 0.946, 0.983, and 0.897, respectively, while Vs Median were 0.970, 0.927, 0.963, 0.941, 0.975, and 0.895, respectively (*p <*0.001). The ROC curves of both E Median (AUC=0.976) Vs Median (AUC=0.970) suggested the remarkable diagnostic efficiency in distinguishing benignity between suspected malignant LNs.

**Conclusions:**

Above results indicated that conventional US together with 2-D SWE could elevate the diagnostic performance. Meanwhile, the parameters of 2-D SWE including E Median and Vs Median could effectively assess malignant LNs, which provide valuable differentiating information in superficial enlarged LNs.

## Introduction

Superficial enlarged lymph nodes (LNs) may serve as a weathervane of various diseases, which further exert great value in evaluating prognosis and guiding treatment. Screening LN status has long been applied clinically, however, conventional imaging modalities such as computed tomography (CT) and magnetic resonance imaging (MRI) are often limited by low accuracy in assessing LNs (1).

Ultrasound (US), as the priority in superficial LN evaluation, could provide pivotal details related to LN characteristics. B-mode US as well as color Doppler US together have indicated specific node architecture and vessel distribution in differentiating benign from malignant (2–4). However, features of different LNs vary from node to node, while overlapped performances of LN have hindered accurate assessment of conventional US, leaving unsatisfactory sensitivity and specificity in cervical LN detection (5). Besides, an unnecessary biopsy may further exacerbate the anxiety of patients (6). Hence, it is crucial to apply reliable techniques especially in detecting superficial enlarged LNs.

Originating in 2003, elastography has been considered a noteworthy modality in ultrasound detecting technologies. Since tumor tissue has been proven to be stiffer than normal tissue, the idea was introduced and optimized into ultrasound diagnostic systems to improve the accuracy in early cancer detection. Substantially, elastography includes strain elastography (SE) and shear-wave elastography (SWE), for which the main difference lies in manual compression vibration (SE) or ultrasound supplied vibration (SWE) (7). It is noteworthy that in SWE, the shear waves generated from acoustic radiation in tissues could be accepted by the ultrasound tracking equipment, thus the elastic modulus (E value) and speed of shear-wave velocity (SWV) of the tissue can both be measured, presenting as kilopascals (kPa) and meters per second (m/s), respectively (8, 9). For clinical application especially, SWE has been extensively applied in thyroid (10, 11), breast (12, 13), liver (14, 15), and other organs (16–18) due to its effective diagnosis through qualitative and quantitative evaluation of tissue stiffness using color map and SWV (1). Featured with non-invasive and quick operating, it could be applied especially in LN detection. Previous studies have indicated that stiffness plays a vital role in differentiating benign from malignant LNs (19). Serving as the trendsetter for systemic cancer metastasis, superficial enlarged LNs usually involve the entire body. However, research related to SWE in LN diagnosis has mostly concentrated on neck or axillary LNs, and the SWV index has rarely been exploited in LN detection.

Hence, our aim of this study is to first investigate the diagnostic efficiency of 2-D SWE in the final diagnosis of superficial enlarged LNs and, second, to compare the diagnostic performance between SWE and conventional US, hoping to add value in determining SWE performance compared with reported studies.

## MaterialS and Methods

### Patients

This retrospective study was conducted from March 2020 to March 2021, among which 204 cases of enlarged superficial LNs were enrolled, including 111 males and 93 females, and ages ranging from 4 to 78 years (mean age 46.0 ± 15.2 years). All LN imaging was performed with conventional US (B-mode and color Doppler mode) and SWE, simultaneously or separately. Inclusion criteria: (i) all LNs were examined before histological biopsy; (ii) LNs that were considered suspicious according to both conventional US and SWE were finally confirmed by histological biopsy results. Exclusion criteria: (i) suspicious LNs with no pathology or unclear pathology results; (ii) LNs with no satisfactory SWE images obtained (e.g., some soft or hard tissue structures around LNs could cause artifacts, which may result in unclear SWE images). The study was approved by the Ethical Committee of General Hospital of Ningxia Medical University. All patients were required to sign written informed consent.

### Methods

#### Conventional US and SWE Examination

All patients were examined primarily by conventional US. Specifically, LNs with a long axis larger than 0.7 cm were considered as superficial enlarged in this study (9). During the process, indicators including size, present or absent of lymphatic hilum, longitudinal axis verses transverse axis ratio (L/T ratio), echogenicity, and color Doppler pattern were evaluated. The examination was performed by Aplio i800 (Canon, Japan) and Acuson sequoia (Siemens, Germany), which were equipped with the i18LX5 linear-array transducer (5-18 MHz) and 10L4 linear-array transducer (4-10 MHz), respectively.

According to WFUMB (7) and EFSUMB (20) Guidelines, SWE could be generally conducted by both point shear-wave elastography (pSWE) and two-dimensional shear-wave elastography (2-D SWE), in which the former is mainly performed by a fixed small region of interest (ROI) to acquire the desired measurement, while the latter uses a larger ROI or more ROIs to realize the “real-time” technique. In the present study, 2-D SWE was adopted. During examination, the same probe conducted in conventional US procedure was kept perpendicular to the patient’s skin with complete exposure of the examination site. After the images were frozen, the elastic modulus value of LNs was measured. The ROI was selected by the default elastic sampling frame. Elastographies are color-coded to represent the elasticity of different tissues, with blue and red indicating the softness and hardness of the tissue, respectively. The elasticity values include the E Median and Vs Median parameters of the elasticity modulus. The measurements were repeated three times for the same LN and averaged. The detecting process of conventional US and 2-D SWE was performed by two ultrasound physicians with at least 5 years of diagnostic experience. The images of both benign and malignant LNs were recorded.

For indeterminate or suspicious LNs, an ultrasound-guided core-needle biopsy was performed, based on the following characteristics: absence of lymphatic hilum, longitudinal versus transverse ratio (L/T ratio) < 2, heterogeneous echogenicity, peripheral or mixed vascularity, and/or E Median ≥ 30 kPa and/or V Median ≥ 3 m/s. LNs went through histopathologic diagnoses as the gold standard by two senior pathologists who were blinded to conventional US and SWE results, and the results were then evaluated.

### Statistical Analysis

Data were analyzed using SPSS software (version 23.0). The data were calculated by chi-square test or t-test. Confidence intervals (CIs) were used as two-sided exact binomial 95% CIs. Sensitivity, specificity, positive predictive value (PPV), and negative predictive value (NPV) were both calculated to determine the diagnostic accuracy. Receiver operating characteristic (ROC) curves were performed to assess the area under the curve (AUC) of E Median and Vs Median. For all data mentioned, *p <*0.05 was considered statistically significant.

## Results

### General Information

**Figure 1:**
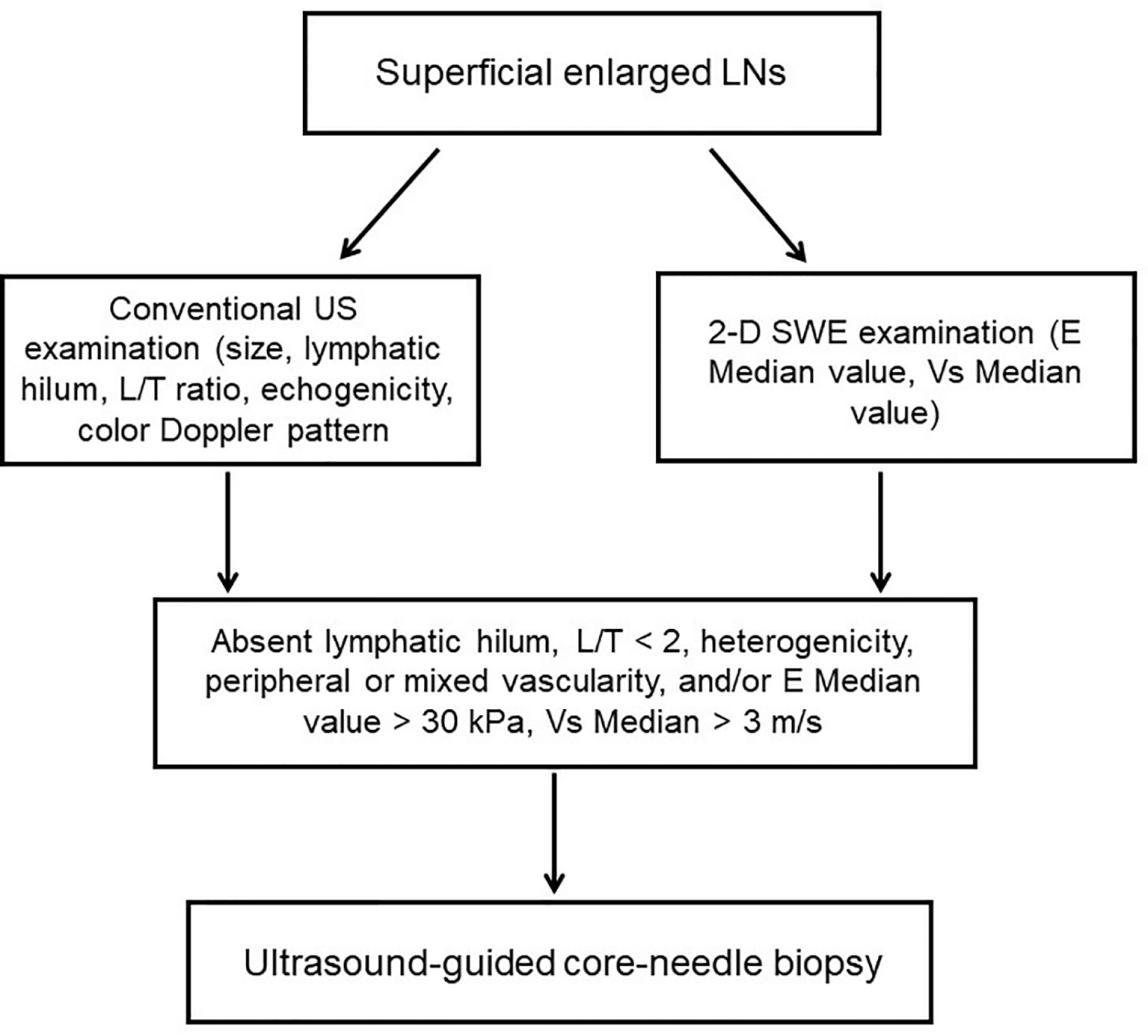
Flowchart of the study. LNs, lymphatic nodes; L/T ratio, longitudinal axis/transverse axis ratio.

Among the 204 cases, there were 111 males and 93 females, with a mean age of 46.0 ± 15.2 years. Of all LNs, 80 were benign LNs, 98 were metastatic LNs, and the rest of the 26 were lymphoma. Among benign LNs, there were 10 tuberculous LNs, 22 nonspecific lymphadenitis, and the rest of the 48 were reactive LNs. The flowchart is demonstrated in **Figure 1**. As results showed, malignant LNs were associated with males and elevated age (**Table 1**).

**Table 1:** General data and conventional US characteristics of LNs.

Variable	Benign (n = 80)	Metastatic (n = 98)	Lymphoma (n = 26)	*p*-value
Age (years)^*^	32.74 ± 9.79	56.24 ± 10.50	48.23 ± 12.38	< 0.01
Gender				
Female (%)	42 (52.5)	37 (37.8)	14 (53.8)	
Male (%)	38 (47.5)	61 (62.2)	12 (46.2)	0.039
Size (mm)^*^				
Longitudinal axis	22.51 ± 4.66	21.51 ± 5.62	20.44 ± 5.06	0.377
Transverse axis	12.14 ± 3.16	17.42 ± 4.55	14.36 ± 3.45	< 0.01
Lymphatic hilum				
Present	68 (85.0)	8 (8.2)	3 (11.5)	
Absent	12 (15.0)	90 (91.8)	23 (88.5)	< 0.001
L/T ratio				
≥ 2	61 (76.2)	2 (2.0)	3 (11.5)	
< 2	19 (23.8)	96 (98.0)	23 (88.5)	< 0.001
Echogenicity				
Homogeneous	77 (96.3)	5 (5.1)	0 (0)	
Heterogeneous	3 (3.7)	93 (94.9)	26 (100)	< 0.001
Color Doppler pattern				
Hilar or central	68 (85.0)	0 (0)	0 (0)	
Peripheral or mixed	12 (15.0)	98 (100)	26 (100)	< 0.001

*Values are presented as the mean ± standard deviation or percentage, respectively.

### Conventional US Evaluation

Conventional US characteristics of all LNs are exhibited in **Table 1**. Results revealed that variables including transverse axis size, lymphatic hilum, L/T ratio, echogenicity, and color Doppler pattern of benign, metastatic LNs and lymphoma were considered with significant differences (*p* < 0.05).

### 2-D SWE Evaluation

**Figure 2:**
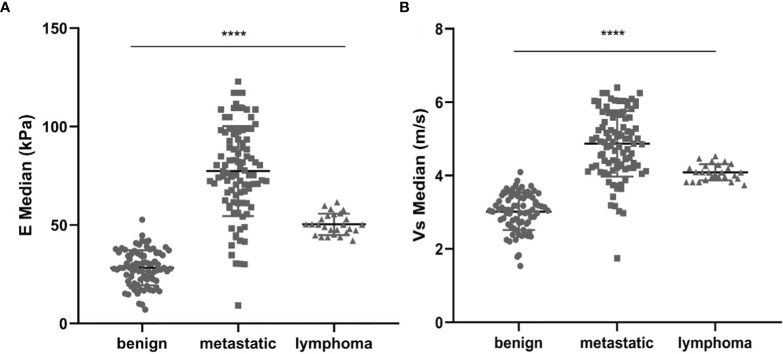
Comparison between E Median values **(A)** and Vs Median values **(B)** of both benign LNs, metastatic LNs, and lymphoma, ****represents *p* < 0.001.

**Figure 3:**
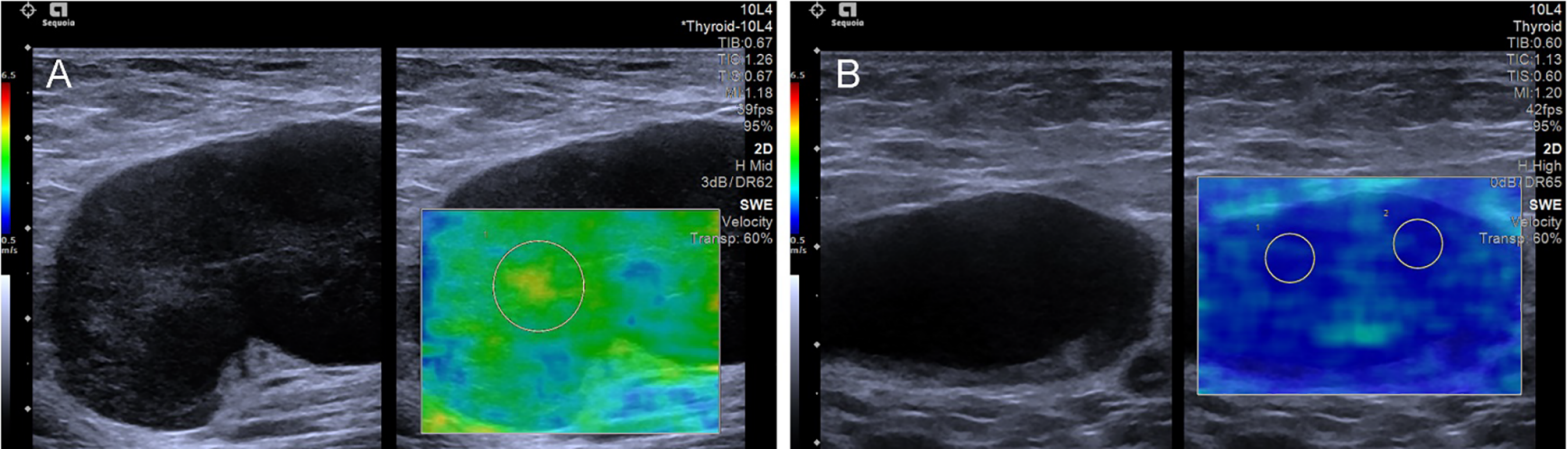
The SWE images of benign LNs, with pathological results proven tuberculous LN **(A)** and nonspecific lymphadenitis **(B)** respectively.

**Figure 4:**
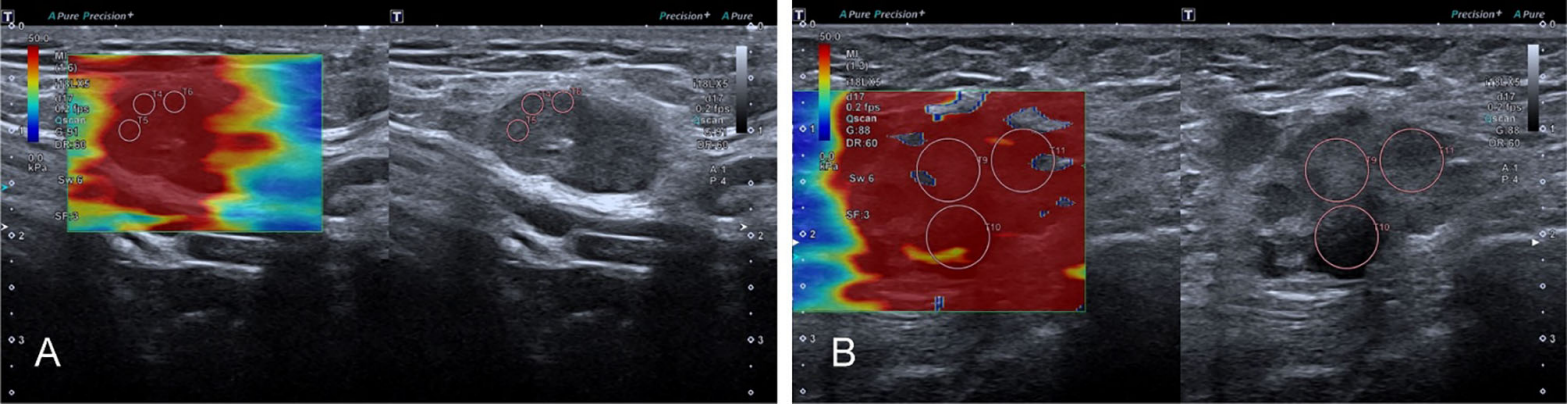
The SWE images of malignant LNs, with pathological results proven metastatic LN in the right axilla **(A)** and metastatic LN in the left axilla of breast cancer **(B)**, respectively.

The mean value of E Median in various LNs are exhibited in **Table 2**, including the values of 28.26 ± 8.87 kPa (benign LNs), 77.46 ± 22.85 kPa (metastatic LNs), and 50.37 ± 5.41 kPa (lymphoma), respectively (*p* < 0.001). The mean value of that in Vs Median were 3.02 ± 0.50 m/s, 4.87 ± 0.90 m/s, and 4.09 ± 0.22 m/s, respectively (*p* < 0.001). SWE Parameters of different types of LNs are also demonstrated in **Figure 2**. The SWE and gray-scale US images are shown in **Figures 3**, **4**.

**Table 2:** SWE parameters of LNs.

Variable	Benign (n = 80)	Metastatic(n = 98)	Lymphoma (n = 26)	*p*-Value
E Median (kPa)^*^	28.26 ± 8.87	77.46 ± 22.85	50.37 ± 5.41	< 0.001
Vs Median (m/s)^*^	3.02 ± 0.50	4.87 ± 0.90	4.09 ± 0.22	< 0.001

*Values are presented as the mean ± standard deviation.

### Comparison of Diagnostic Performance Between Conventional US and SWE

According to pathological results, the diagnostic performance of conventional US, 2-D SWE, and the two combined were compared. Specifically, the sensitivity, specificity, PPV, NPV, and overall accuracy of conventional US were 0.902, 0.825, 0.856, 0.880, and 0.727, respectively, while that of SWE revealed elevated results of 0.911, 0.863, 0.903, 0.873, and 0.773, respectively, and the two combined revealed the highest of all indicators among the groups (**Table 3**).

**Table 3:** Diagnostic performance of Conventional US, SWE, and Conventional US combined SWE.

Method	Sensitivity (95% CI)	Specificity(95% CI)	PPV(95% CI)	NPV(95% CI)	Overall Accuracy
Conventional US	0.902 (0.818~0.952)	0.825 (0.720~0.898)	0.856 (0.766~0.916)	0.880 (0.779~0.940)	0.727
SWE	0.911 (0.838~0.954)	0.863 (0.763~0.926)	0.903 (0.829~0.948)	0.873 (0.775~0.934)	0.773
Conventional US + SWE	0.932 (0.852~0.972)	0.887 (0.785~0.947)	0.911 (0.828~0.958)	0.913 (0.814~0.964)	0.819

CI, confidence intervals; PPV, positive predictive value; NPV, negative predictive value.

### Diagnostic Value of 2-D SWE for Predicting Malignant LNs

As seen in **Table 4**, the optimal cutoff value of E Median and Vs Median were 42.90 kPa and 3.73 m/s, respectively. As the table shows, AUC value, sensitivity, specificity, accuracy, PPV, and NPV of E Median were 0.976, 0.927, 0.975, 0.946, 0.983, and 0.897, respectively, and that of Vs Median were 0.970, 0.927, 0.963, 0.941, 0.975, 0.895, respectively (*p <*0.001).

**Table 4:** Diagnostic performances of SWE examined malignant LNs.

Variable	Cutoff value	AUC(95% CI)	Sensitivity(95% CI)	Specificity (95% CI)	Accuracy	PPV	NPV	*p*-Value
E Median (kPa)	42.90	0.976 (0.955~0.996)	0.927 (0.863~0.964)	0.975 (0.904~0.996)	0.946	0.983	0.897	< 0.001
Vs Median (m/s)	3.73	0.970 (0.946~0.993)	0.927 (0.863~0.964)	0.963 (0.887~0.990)	0.941	0.975	0.895	< 0.001

kPa, kilopascal; AUC, area under the ROC curve; CI, confidence intervals; PPV, positive predictive value; NPV, negative predictive value. kPa, kilopascals; m/s, meters per second.

**Figure 5:**
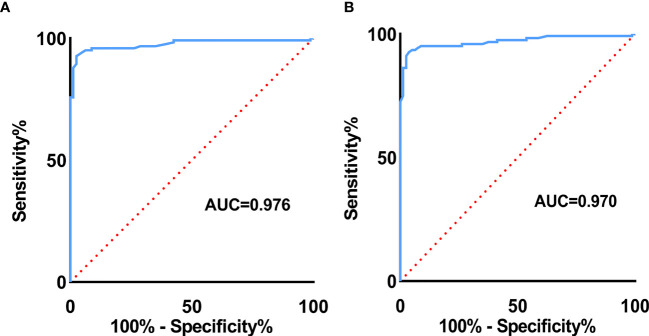
ROC curve of E Median value **(A)** and Vs Median value **(B)** for malignant LN prediction.

To explore the diagnostic efficiency of SWE, the ROC curve was performed. As revealed in **Figure 5**, the ROC curve of E Median (AUC=0.976) suggested a significant value in distinguishing benignity between malignancy of LNs, and the same ROC curve of Vs Median (AUC=0.970) is also demonstrated (**Figure 5** **)**.

## Discussion

It is well acknowledged that the accurate assessment of LNs preoperatively may greatly affect further treatment and prognosis of patients (21). Hence, appropriate modality plays a key role in assessing LN status. In the present study, 2-D SWE, and conventional US were performed for differentiating superficial enlarged LNs. The results indicated that SWE had better diagnostic performance compared with conventional US when applied alone, while the two combined exhibited the highest diagnostic efficiency. More importantly, AUC and cutoff value of E Median and Vs Median all demonstrated that SWE could serve as an effective tool in differentiating superficial enlarged LNs.

Previous research has acknowledged that conventional US is the most commonly applied modality in LN detection. Our study showed that indicators such as lymphatic hilum absence, L/T ratio < 2, heterogeneous echogenicity, and peripheral or mixed vascularity were more likely to be associated with malignant features. Distribution of vascularity in LN is essential for differentiating LN status, however, it is generally accepted that benign LNs such as reactive hyperplastic LNs may also indicate not-so-typical hilar or central vascularization (9, 22). Results of our study are in line with these opinions. SWE, however, uses subjective parameters to reflect tissue stiffness.

In our study, both the E Median and Vs Median of malignant LNs (metastatic LNs and lymphoma) were remarkably higher than that of benign ones, and those of metastatic LNs were among the highest, which share similarities with previous studies (23–25). Since the invasiveness of malignancy may lead to the deposited extracellular matrix and cross-linked collagen to finally affect the stiffness of tissue growth (21, 26), besides, calcification and inflammation of lesions may also elevate tissue stiffness (27). Hence, the metastatic LNs may also become keratinized due to tumor infiltration and interstitial proliferation. Since tumor growth and invasion rely greatly on fibrosis through a whole series of regulating factors, it is reasonable to understand the increase of tissue stiffness both in the tumor and its metastatic LNs. Lymphoma in our study exhibited a medium level of both E Median and Vs Median values, which is in line with previous studies (24, 28). However, Yang et al. (9) indicated a cutoff value of 31.6 kPa in the stiffest region with an accuracy of 65.2%, sensitivity of 55.95%, and specificity of 96%, and Wen et al. (29) demonstrated an Emax value of 66 kPa for cervical metastatic LN, which was lower than our study. The reasons may be because, firstly, different equipment and different sites of LNs may result in differentiated SWE values. Meanwhile, despite calcification in enlarged lymphomas, the higher chance of calcification in tuberculous LNs may also result in a corresponding increase in E value, which might be one of the reasons for false-positive results. Chen et al. indicated that in tuberculous LNs, the epithelioid cells and multinucleated giant cells would proliferate to induce normal structured LNs substituted by small foci, and the cutoff value of Emax was 85 kPa in the study (30). Furthermore, some of the reactive hyperplasia in benign LNs is caused by various acute and chronic inflammation. In acute inflammation, the internal tissue structure of LNs is not destroyed, thus leading to a lower E value, while chronic inflammation may reveal a higher E value due to fibrosis in the nodes.

Despite of E Median, SWV is also considered one of the parameters related to tissue stiffness. In this study, a total of 204 lymph nodes were included, of which 80 were benign LNs, 98 were metastatic LNs, and 26 were lymphoma. The results of average SWV (Vs Median) indicated that benign LNs exhibit a much lower Vs Median value (3.02 ± 0.50 m/s) compared with malignant ones (4.87 ± 0.90 m/s) and lymphoma (4.09 ± 0.22 m/s). According to Rüger et al. (24), different lesions of stiffness exhibited a significantly different expression of SWV, with benign LNs ranging from 2.7 ± 0.8 to 4.1 ± 0.1.8 m/s, while the malignant ones ranged from 4.1 ± 0.1.2 to 8.3 ± 1.7 m/s (24). The main difference may be because, firstly, Rüger et al. excluded lymphoma in the study, which they considered softer than malignant LNs; secondly, they performed the virtual touch imaging quantification (VTIQ) technique, which may result in different SWV outcomes. Cai et al. also showed that SWV values in malignant nodes were remarkably higher than that of benign ones (31). The reason may be because hyperplastic lymphoid tissue often leads to an increased size of benign LNs, which in turn would result in a relatively low SWV. In metastatic lymph nodes, due to tumor invasion, deposition, and proliferation, the tissue stiffness in LNs varies greatly, and the tissue in the sampling frame area becomes less displaced under the acoustic beam, which may lead to an increased dissemination speed and elevated Vs Median (32).

In our study, the cutoff value suggested 42.90 kPa for predicting malignant LN as the optimal threshold of E Median, which is much higher than previous studies (33), while in accordance with the research of Desmots et al. (34). Besides, our study indicated that AUC, sensitivity, specificity, and accuracy in E Median and Vs Median were both higher than in previous studies. The reasons may be summarized as follows: firstly, the stiffness of target LNs varies according to their pathological period and different primary tumors (35); secondly, the stiffness is closely related to the anatomical features of the body where the target LNs are located. Hence, the uneven skin of the neck and the presence of the large blood vessels or trachea may result in a higher E Median value, while axillary with loose subcutaneous tissue may indicate a lower value, and the reason is still unclear (25). In this study, the diagnostic performance of both conventional US, 2-D SWE, and the two combined showed that although SWE indicated an elevated diagnostic efficiency, the two combined were still higher than that of the single performance. This result suggested that combined evaluations should be adopted in the clinical practice to evaluate suspicious LNs, which was in line with previous research (35, 36).

Our study has some limitations. Firstly, it was a retrospective study, and selection bias may be unavoidable. Secondly, samples in our study were small and comprehensive explorations are still needed in the future. Thirdly, since we have chosen two different ultrasound systems for SWE detection, there may be slight differences in the results.

In conclusion, our result indicated that 2-D SWE combined with conventional US could reveal an elevated diagnostic efficiency in superficial enlarged LN detection. Besides, the increased SWE parameters were closely related to malignancy in superficial enlarged LN assessment, suggesting it could be applied as a promising imaging method of differentiating benign and malignant LNs preoperatively. As the complementary modality in LN detection, SWE exerts unique advantages for malignant LNs which may further provide vital information on disease prognosis.

## Data Availability Statement

The original contributions presented in the study are included in the article/supplementary material. Further inquiries can be directed to the corresponding authors.

## Ethics Statement

The studies involving human participants were reviewed and approved by Scientific Research Ethics Committee of General Hospital of Ningxia Medical University. Written informed consent to participate in this study was provided by the participants’ legal guardian/next of kin. Written informed consent was obtained from the individual(s), and minor(s)’ legal guardian/next of kin, for the publication of any potentially identifiable images or data included in this article.

## Author Contributions

YS and WW contributed equally to this work. YS and WW collected the data, QZ wrote this paper, and KZ and CM revised this manuscript. All authors contributed to the article and approved the submitted version.

## Funding

This work was supported by the National Outstanding Youth Science Fund Project of National Natural Science Foundation of China (Grant Number: 82022033), Shanghai Rising-Star Program (Grant No. 19QA1406800), Shanghai Talent Development Fund (Grant No. 2019040), the Fundamental Research Funds for the Central Universities (22120210561) and the program for Shanghai Young Top-Notch Talent.

## Conflict of Interest

The authors declare that the research was conducted in the absence of any commercial or financial relationships that could be construed as a potential conflict of interest.

## Publisher’s Note

All claims expressed in this article are solely those of the authors and do not necessarily represent those of their affiliated organizations, or those of the publisher, the editors and the reviewers. Any product that may be evaluated in this article, or claim that may be made by its manufacturer, is not guaranteed or endorsed by the publisher.
